# The chordate ancestor possessed a single copy of the *Brachyury* gene for notochord acquisition

**DOI:** 10.1186/s40851-017-0064-9

**Published:** 2017-03-23

**Authors:** Jun Inoue, Yuuri Yasuoka, Hiroki Takahashi, Noriyuki Satoh

**Affiliations:** 1 Marine Genomics Unit, Okinawa Institute of Science and Technology Graduate University, Onna, Okinawa, 904-0495 Japan; 2 Developmental Biology, National Institute for Basic Biology, Nishigonaka 38, Myodaiji, Okazaki, Aichi 444-8585 Japan

**Keywords:** *Brachyury*, Primary and secondary expression domains, Blastopore, Notochord, Gene duplication, Chordate evolution

## Abstract

**Background:**

The T-box family transcription-factor gene, *Brachyury*, has two expression domains with discrete functions during animal embryogenesis. The primary domain, associated with the blastopore, is shared by most metazoans, while the secondary domain, involved in the notochord, is specific to chordates. In most animals*, Brachyury* is present in a single copy, but in cephalochordates, the most basal of the chordates, the gene is present in two copies, suggesting allotment of the two domains to each of the duplicates.

**Results:**

In order to clarify whether *Brachyury* duplication occurred in the common ancestor of chordates after which one of duplicates was lost in the urochordate and vertebrate lineages, we estimated phylogenetic relationships of *Brachyury* genes and examined the synteny of a *Brachyury*-containing genomic region of deuterostomes with decoded genomes*.* The monophyletic origin of tandemly arranged *Brachyury* genes of cephalochordates indicates that the tandem duplication occurred in the cephalochordate lineage, but not in the chordate ancestor.

**Conclusions:**

Our results thus suggest that, in the common ancestor of chordates, a single copy of *Brachyury* acquired two expression domains and that the duplication was not involved in the acquisition of the notochord. However, in relation to regulatory mechanisms, both possibilities—namely a single copy with two domains and two copies with different domains—should be considered in future studies of *Brachyury*.

**Electronic supplementary material:**

The online version of this article (doi:10.1186/s40851-017-0064-9) contains supplementary material, which is available to authorized users.

## Background

We are interested in genetic mechanisms involved in the origins and evolution of chordates [1]. Chordates comprise three taxa, cephalochordates, urochordates or tunicates, and vertebrates [2]. These are thought to have originated from a common ancestor of the deuterostomes, together with ambulacrarians, a clade containing echinoderms and hemichordates. The organ that best characterizes chordates is the notochord, an organ that supports the beating of the muscular tail of fish-like larvae or adults [1, 3, 4]. The T-box family transcription-factor gene, *Brachyury*, plays an essential role in notochord formation [5]. In ascidians (urochordates), for example, *Brachyury* is expressed exclusively in primordial embryonic notochord cells [6, 7]. Loss of *Brachyury* function results in the failure of notochord formation, while its ectopic expression induces endoderm cells to become notochord cells [8, 9].

Interestingly, *Brachyury* is not specific to chordates, but is present in most metazoans, including non-chordate deuterostomes [5, 10]. Beside paralogs derived from ancient whole genome duplication (referred to as “ohnologs”), *Brachyury* is usually present as a single copy, with some exceptions (*Hydra* [11], calcisponge [12], and cephalochordates (see below)), and is expressed around the blastopore during gastrulation. A recent study by our group using coral embryos demonstrated that the evolutionarily conserved function of *Brachyury* is associated with formation of blastopore-derived organs, such as the pharynx of coral embryos [13]. In non-chordate deuterostomes, ambulacrarians, *Brachyury* is expressed in the archenteron invagination region in early gastrulae and in the stomodeum invagination region in later stage embryos [14, 15]. Although an overexpression experiment with sea urchin *Brachyury* suggested its role in gastrulation [16], the developmental role of ambulacrarian *Brachyury* remains to be determined.

We proposed an evolutionary scenario for *Brachyury* emphasizing its primary and secondary expression domains and functions [5]. Namely, ambulacrarians require *Brachyury* in its primary domain of expression and function associated with the blastopore (PEF), while chordates employ the gene, not only for PEF, but also for the secondary domain of expression and function associated with the notochord (SEF). In urochordates, the PEF was likely lost due to the precocious mode of embryogenesis [1, 5]. For the past decade, evolutionary developmental biologists have been asking how the chordate ancestor acquired the *Brachyury* SEF. Answering this question is critical to our understanding of genetic and molecular mechanisms involved in the origins of chordates. Genomes of the cephalochordates, *Branchiostoma floridae* and *B. belcheri*, each contain a set of duplicated *Brachyury*, *Amphi-Bra1* and *Amphi-Bra2* [17, 18]. These duplicated genes have both PEF and SEF, but the expression domains of each have not been determined [17, 19]. In contrast, urochordates have only a single copy [20]. Most vertebrates also have a single copy of *Brachyury*/*T*, although they have variable numbers of *T* ohnologs, which arose from the two rounds of genome-wide gene duplication (2R-GWGD) that occurred in this lineage [21, 22]. In mice, only a *T* gene exhibits PEF and SEF during early embryogenesis [22].

**Fig. 1:**
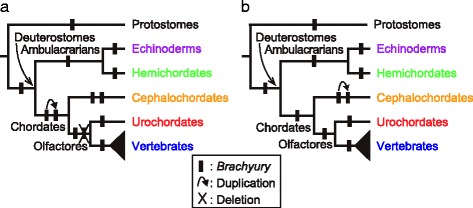
Two alternative evolutionary scenarios for *Brachyury* in relation to gene duplication along with diversification of five deuterostome lineages. **a** In the first scenario, *Brachyury* duplicated in a common ancestor of chordates, with an original gene copy retaining the primary expression-function (PEF) while the duplicate developed the secondary expression-function (SEF). The cephalochordate lineage retains this feature while the urochordate and vertebrate lineages lost one of duplicates in their last common ancestor. *Brachyury* evolution in vertebrates is complex, due to the 2R-GWGD that occurred in this lineage. **b** In the second scenario, chordates maintained a single-copy of *Brachyury*, just as non-chordate deuterostomes did. Duplication occurred only in the lineage leading to cephalochordates, but not in lineages leading to urochordates and vertebrates

The occurrence of two domains of *Brachyury* expression and function may be explained by one of two alternative evolutionary scenarios (Fig. 1). In scenario 1, *Brachyury* was present as a single copy in deuterostome ancestors, while it became duplicated in an ancestor of chordates, such that the original gene retained PEF and its newly formed counterpart obtained SEF (Fig. 1a). The cephalochordate lineage retained this arrangement, whereas the urochordate and vertebrate lineages lost one of duplicates. In scenario 2 (Fig. 1b), chordates retained a single copy of *Brachyury* as did the non-chordate invertebrates. Duplication occurred in the lineage leading to cephalochordates, but not in the lineage leading to olfactores (urochordates + vertebrates). Evolution of vertebrate T-box family genes seems to have been complicated by the 2R-GWGD, and vertebrate *Brachyury*/*T* needs to be examined more carefully in the future (Yasuoka et al., in preparation).

In 2015, the genomes of two hemichordate species, an indirectly developing acorn worm, *Ptychodera lava*, and a directly developing acorn worm, *Saccoglossus kowalevskii*, were decoded [23]. Genomes of animals belonging to each of the four other phyla were already decoded prior to 2014; echinoderm sea urchin (*Strongylocentrotus purpuratus*; [24]) and starfish (*Acanthaster planci* [25]), cephalochordate amphioxus (*Branchiostoma floridae* [26] and *Branchiostoma belcheri* [27]), urochordates (e.g., *Ciona intestinalis* [20] and *Oikopleura dioica* [28]) and vertebrates (e.g., *Homo sapiens* [29]). We obtained genomic information for all five phyla of deuterostomes, enabling us to examine which of the two scenarios outlined above better explains the genetic and molecular evolution of *Brachyury* in relation to chordate origins.

## Methods

Gene trees were estimated with an analytical pipeline implementing BLAST search and the maximum likelihood method (modified from Inoue et al. [30]).

### BLAST search

Human and lancelet protein-coding sequences (amino acids) were used as queries for BLASTP search [31] against all protein-coding sequences in 18 selected animal genomes (Table 1). The resulting BLAST top 20 hits were screened using an E-value cutoff of <10^−3^ [32]. Where transcript variants existed for a single locus, only the longest sequence was used in the present analysis.

**Table 1:** List of bilaterian species with decoded genomes used in this study

Species	*Brachyury* genes	
	Gene name	Gene/Protein ID
Protostomia		
Ecdysozoa		
*Drosophila melanogaster* ^a^	*brachyenteron*	FBpp0304380
*Caenorhabditis elegans* ^a^	—	—
Lophotrochozoa		
*Lingula anatina* ^a^	*brachyury*	g6294
*Octopus bimaculoides* ^a^	*brachyury*	Ocbimv22020340m.p
*Lottia gigantea* ^a^	*brachyury*	LotgiP154800
*Crassostrea gigas* ^a^	*brachyury*	EKC28765
Deuterostomia		
Ambulacraria		
Hemichordata		
*Ptychodera flava* ^b^	*brachyury*	g18670
*Saccoglossus* ^b^	*brachyury*	Sakowv30011577
*kowalevskii*		
Echinodermata		
*Strongylocentrotus*	*brachyury*	SPU_013015
* purpuratus* ^a^		
*Acanthaster planci* ^c^	*brachyury*	oki15-190
Chordata		
Cephalochordata		
*Branchiostoma floridae* ^d^	*Amphi-Bra1*	279431
	*Amphi-Bra2*	121413
*Branchiostoma belcheri* ^e^	*Amphi-Bra1*	102780R
	*Amphi-Bra2*	102770 F
Urochordata		
*Oikopleura dioica* ^f^	*brachyury*	GSOIDT00000279001
*Botryllus schlosseri* ^g^	*brachyury*	g63408
*Ciona intestinalis* ^h^	*brachyury*	ENSCINP00000001477
*Ciona savignyi* ^h^	*brachyury*	ENSCSAVP00000003798
Vertebrata		
*Gallus gallus* ^h^	*brachyury/T*	ENSGALP00000018703
	*tbx19*	ENSGALP00000024551
*Homo sapiens* ^h^	*brachyury/T*	ENSP00000296946
	*TBX19*	ENSP00000356795

^a^EnsemblMetazoa [43]

^b^Hemichordate Genomes [44]

^c^MarinegenomicsDB [45]

^d^Branchiostoma floridae-JGI Genome Portal [46]

^e^LanceletDB [47]

^f^OikoBase [48]

^g^Botryllus schlosseri Genome Project [49]

^h^Ensembl79 [50]

### Alignment

The sequences of proteins obtained by the BLASTP search were aligned using MAFFT [33]. Multiple sequence alignments were trimmed by removing poorly aligned regions using TRIMAL 1.2 [34] with option “gappyout.” Corresponding cDNA sequences were forced onto the amino acid alignment using PAL2NAL [35] to generate nucleotide alignments for later comparative analysis. Each gene sequence was checked, and removed from the alignment as spurious BLAST hits if the sequence was shorter than 55% of the length of the query sequence in the unambiguously aligned sites.

### Gene tree search

Phylogenetic analyses were conducted by the maximum likelihood method aligned with bootstrap analysis based upon 100 replicates. The first and second codon positions were used for DNA analysis. The analysis was performed by RAxML 8.2.4 [36], which invokes a rapid bootstrap analysis and search for the best scoring ML tree. The GTRGAMMA (general time-reversible [37] with the gamma [38]) and the PROTGAMMAWAGF (WAG [39] with gamma and empirical base frequencies) models were used for DNA and amino acid analyses, respectively. The synteny of a *Brachyury*-containing genomic region was assessed by identifying human/*Drosophila* ortholog of each neighboring gene by estimating each gene tree via our analytical pipeline.

## Results

We examined *Brachyury* of representative protostomes and deuterostomes, the genomes of which have been decoded (Table 1, with Brachyury gene/protein ID and information from the genomes). Phylogenetic relationships of deuterostome species are based on previous studies (e.g., [23]).

### Copy number of *Brachyury* in deuterostomes and the chordate ancestor

Molecular cloning of mouse *Brachyury* was followed by identifying its orthologs in various metazoans (reviewed by [5, 40]). In addition, cloning of other T-box-containing transcription factor genes, including *T-brain*, *Tbx1*, *Tbox2*, and *Tbx6*, shows that they form a family called the T-box family (e.g., [21]). Although *Brachyury* has been identified and characterized in each bilaterian with a decoded genome, we examined *Brachyury* copy numbers in 18 species, including six protostomes and 12 deuterostomes (Table 1). To evaluate the two scenarios (Fig. 1), we did not include ohnologs in vertebrates as the copy number of *Brachyury* because that duplication is not associated with the acquisition of SEF.

To this end, we first carefully identified all T-box containing genes from the decoded genomes. In order to identify a candidate query sequence from non-vertebrate chordate lineages, using the human Brachyury amino acid sequence as a query, phylogenetic relationships were roughly estimated to delineate a clade comprising the Brachyury subfamily. Using queries of human and cephalochordate Brachyury amino acid sequences (Additional file 1), we next searched for the *Brachyury* gene in selected bilaterian genomes by estimating phylogenetic relationships (Additional file 2). Although no ortholog was found in the present genome assembly of the nematode, *Caenorhabditis elegans*, a single copy of *Brachyury* was found in each of the other bilaterians in both resultant trees based on nucleotide and amino acid datasets. An exception was that lancelets (cephalochordates) possessed two copies in their genome, as shown previously [17, 18].

**Fig. 2:**
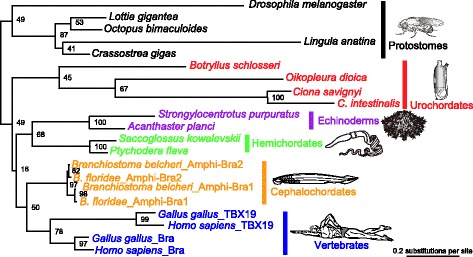
Molecular phylogeny of *Brachyury* family members. The tree was estimated based on a DNA dataset comprising 872 unambiguously aligned sites (excluding 3rd codon positions) of the sequences selected from the estimated gene tree of T-box family members (Additional file 2). Numbers at nodes are bootstrap probabilities obtained for 100 replicates. Because base-pair change is apparently faster in urochordates than in other deuterostomes, urochordate *Brachyury* forms a clade with that of other deuterostomes. It should be noted that the two species of cephalochordate each have two copies of *Brachyury*

In order to estimate the copy number of *Brachyury* in the chordate ancestor, we reconstructed phylogenetic relationships of *Brachyury* using only *Brachyury* gene sequences (Additional file 3) selected from the estimated tree of T-box containing genes (Additional file 2). Estimated gene trees based on the comparison of nuclear (Fig. 2) and amino acid (Additional file 4) sequences produced the same deuterostome relationships, except for the positions of the urochordate, *Botryllus schlosseri* and a clade consisting of the remaining urochordates. For subsequent discussion, we used the tree obtained from DNA analysis because of the longer sequences. The *Brachyury* tree (Fig. 2) differed from the species tree (e.g., [23]) in that, in the *Brachyury* tree, urochordate genes formed a sister clade with all remaining deuterostome genes. This may be the result of a faster evolutionary rate of urochordate proteins than in other deuterostome taxa. Our results (Fig. 2) indicate that, with bootstrap support of 97%, *Amphi-Bra1* and *Amphi-Bra2* of the two species of cephalochordates forms a monophyletic group, consistent with our previous study [19].

Given that cephalochordates are the only deuterostome taxon with the duplicated state of *Brachyury*, and that the two copies form a monophyletic clade (Fig. 2), it is highly likely that the duplication of *Brachyury* was specific to the cephalochordate lineage, and did not occur in the chordate ancestor. Namely *Brachyury* was present in a single copy in the chordate ancestor. Therefore, our results support the second of the two scenarios described above (Fig. 1b). In vertebrates (chicken and human, here), *Brachyury*/*T* forms a clade while *Tbx19* forms another clade (Fig. 2). This suggests that 2R-GWGD resulted in divergence of the ancestral *Brachyury* into a clade including *Brachyury* or *Tbx19*.

### Genomic organization of *Brachyury* in deuterostomes

Next, to gain a better understanding of the evolutionary changes in the genetic and genomic organization of *Brachyury* in relation to chordate evolution, we examined the synteny of genes in *Brachyury*-containing genomic regions, especially in deuterostome taxa. Although global synteny analyses have shown comparable and conserved synteny between cephalochordate and vertebrate genomes [26] and between hemichordate and cephalochordate genomes [23], no detailed analyses were carried out on genomic regions that contain *Brachyury*.

**Fig. 3:**
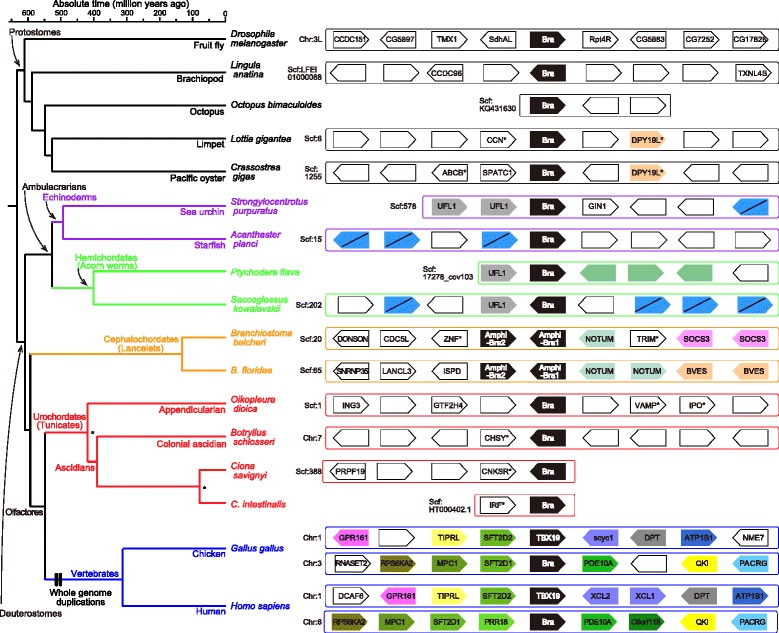
A schematic comparing the genomic organization of *Brachyury* and its neighboring genes within scaffolds/chromosomes. Presence and transcriptional direction of the genes are shown with *boxes*. Boxes of the same color or protein names show orthologous relationships (or paralogous within a given species) while *white boxes* show no orthologous relationship to any known proteins. Note that genes indicated in *blue boxes* with *black slanting lines* share domains with a comparable or similar DNA sequence. Divergence times are based on TIMETREE database [42], except for nodes with asterisks (no estimate)

In the genomes of two cephalochordate species, *B. floridae *and *B. belcheri*, the two *Brachyury* were tandemly aligned (Fig. 3). In both genomes, although a neighboring NOTUM-like gene is present syntenically, synteny of neighboring genes is limited to the four genes. This suggests that the two copies of *Brachyury* arose from a tandem duplication, not from an ancient segmental duplication.

Beside the trait of tandem duplication of cephalochordate *Brachyury*, our synteny analysis characterized conserved synteny around *Brachyury* of major deuterostome lineages (Fig. 3). A global view of synteny indicates that it is comparable among ambulacrarian species, because genes with comparable sequences were found in echinoderms and an acorn worm. On the contrary, no conserved synteny was found among four species of tunicates. Although the synteny analysis among vertebrate genomes was complicated by 2R-GWGD, it is evident that there are no scaffolds with duplicated *Brachyury*/*T* or its ohnolog, *Tbx19*, in chicken or human genomes. Detailed synteny of the *Brachyury*-containing genomic region is discussed in Additional file 5.

## Discussion

*Brachyury* is thought to be the most ancient T-box family member (e.g., [21, 22]). It has been speculated that during their histories of over 500 million years, each of the five deuterostome taxa altered the genetic and genomic constitution of *Brachyury* from its original forms, rending analysis of the present question of *Brachyury* evolution more difficult than that of more recently evolved genes. Nevertheless, the tandem duplicated state of *Brachyury* is found only in cephalochordates. Recently, another example of tandem duplication of chordate *Brachyury* has been reported in an amphibian, *Xenopus tropicalis* [41]. It thus appears likely that cases of *Brachyury* duplication [1] are exceptional. As previously described, *Brachyury* has two domains of expression and function. The primary domain of expression is in the blastopore during gastrulation (PEF), which is shared by all metazoans [5, 13]. The secondary domain is associated with the notochord (SEF), and is specific to chordates. It is tempting to speculate that one is the original *Brachyury* gene with its original function and the other is a new copy with a secondary function, and that the duplication occurred very early in chordate evolution. However, as shown in the present study, *Brachyury* was present as a single copy in the common ancestor of chordates and a single copy of *Brachyury* acquired the secondary function, SEF, in the chordate ancestor.

## Conclusions

The presence of a single copy of *Brachyury* in the chordate ancestor indicates that the gene duplication was not associated with the acquisition of SEF leading to the development of the notochord. Considering that copy numbers of *Brachyury* vary between cephalochordates and other deuterostome lineages, the question of regulatory mechanisms still remains. Intensive analyses have been carried out to elucidate 5′ upstream sequences or modules that regulate PEF and SEF, respectively [1]. Therefore, we have to keep the two cases, namely a single copy with two domains and two copies with different domains, in mind for future studies of *Brachyury* in relation to regulatory mechanisms.

## Additional files

Additional file 1: cDNA sequence alignment of T-box family members analyzed in the phylogenetic analysis (Additional file 2). Identity to one of the query gene sequences (underlined) is denoted by dots. Only unambiguously aligned sites are presented (525 out of 16,677 sites). Insertions/deletions of specific nucleotides are indicated by dashes. (PDF 9491 kb)

Additional file 2: Molecular phylogenies of T-box family members based on a DNA dataset comprising 350 unambiguously aligned sites (excluding 3rd codon positions) (a) and based on an amino acid dataset comprising 175 sites (b). In both trees, the Brachyury family consistently forms a distinct clade among T-box family members. The resulting tree obtained from reanalysis using only this portion is shown in Fig. 2 and Additional file 4. Query sequences used for the BLAST search are marked with black dots. (PDF 551 kb)

Additional file 3: cDNA sequence alignment of *Brachyury* genes (2625 sites in total) analyzed in the phylogenetic analyses (Fig. 2 and Additional file 4). The alignment was constructed using the selected gene sequences from the estimated tree of T-box-containing genes (Additional file 2). Unambiguously aligned sites indicated by a 1 (on the top of the sequences, 1308 sites) were used for the analyses. (PDF 3214 kb)

Additional file 4: Molecular phylogeny of Brachyury family members based on an amino acid dataset comprising 436 unambiguously aligned sites. Arrowheads indicate topological incongruities with the tree obtained from comparisons of nucleotides (Fig. 2). Probably due to the short length of the analyzed sequence, the *Botryllus schlosseri* (urochordate) gene was placed as a sister lineage of a clade comprising cephalochordate and vertebrate genes. (PDF 150 kb)

Additional file 5: Conserved synteny around *Brachyury* in deuterostomes. (DOCX 125 kb)

## Abbreviations

PEF: Primary domain of expression and function associated with the blastopore
SEF: Secondary domain of expression and function associated with the notochord
