# Supplementary material for: The chordate ancestor possessed a single copy of the Brachyury gene for notochord acquisition
Source: Zoological Lett. 2017 Mar 23;3:4. doi: 10.1186/s40851-017-0064-9 (PMC5363035; doi:10.1186/s40851-017-0064-9)
Supplement: Supplementary file 1 — cDNA sequence alignment of T-box family members analyzed in the phylogenetic analysis (Additional file 2). Identity to one of the query gene sequences (underlined) is denoted by dots. Only unambiguously aligned sites are presented (525 out of 16,677 sites). Insertions/deletions of specific nucleotides are indicated by dashes. (PDF 9491 kb) [file 40851_2017_64_MOESM1_ESM.pdf]

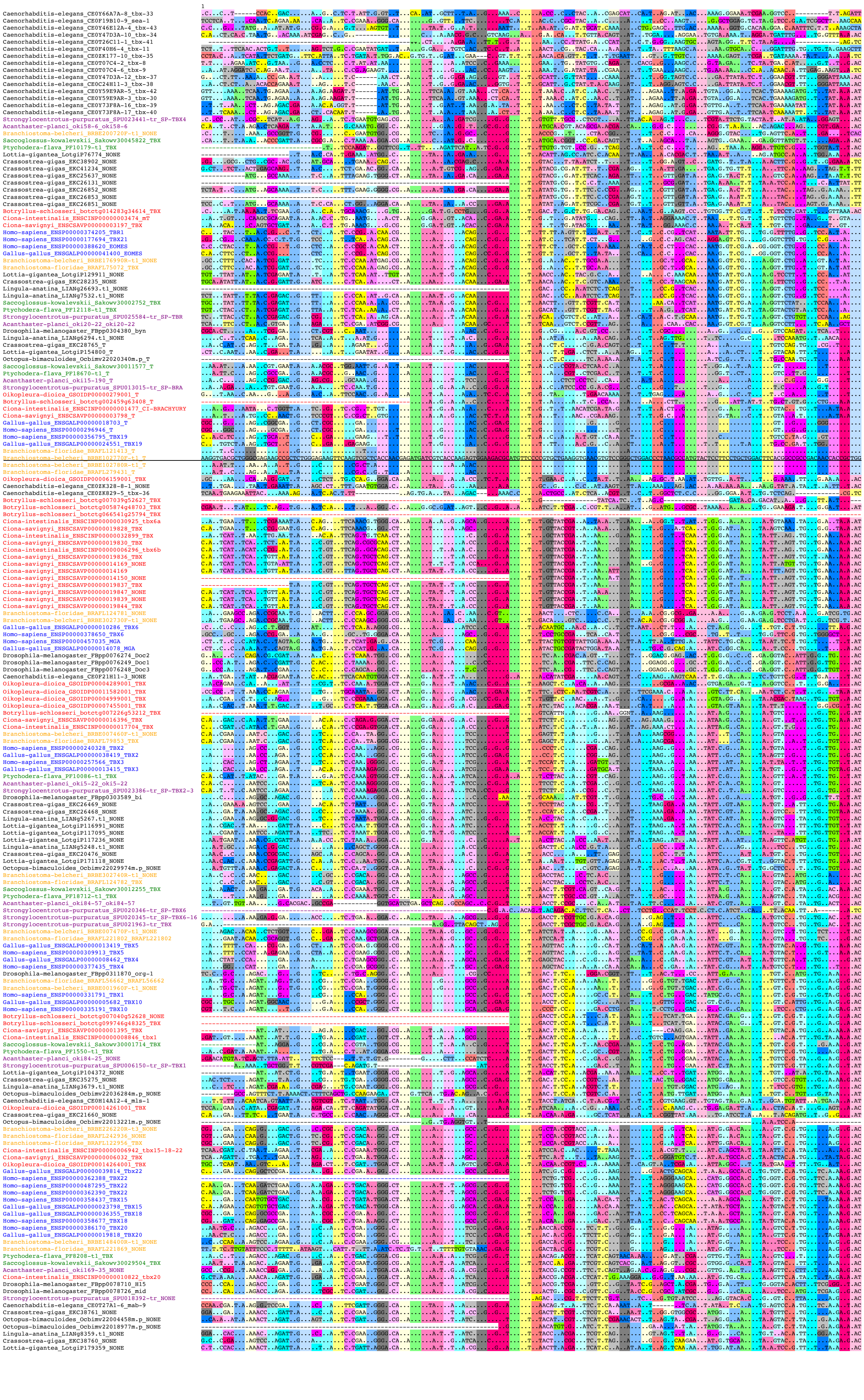

Additional file 1

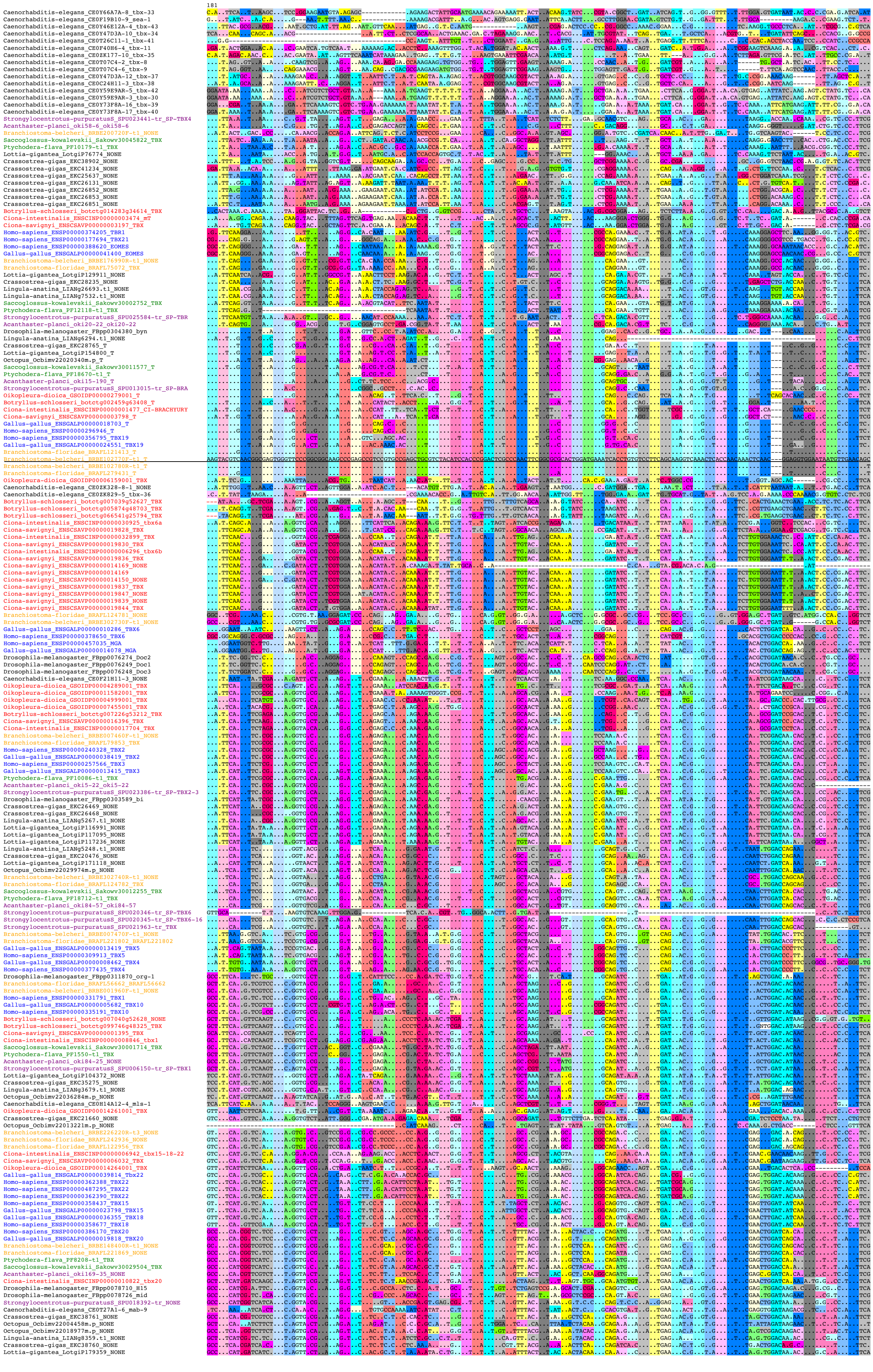

Additional file 1
(continued)

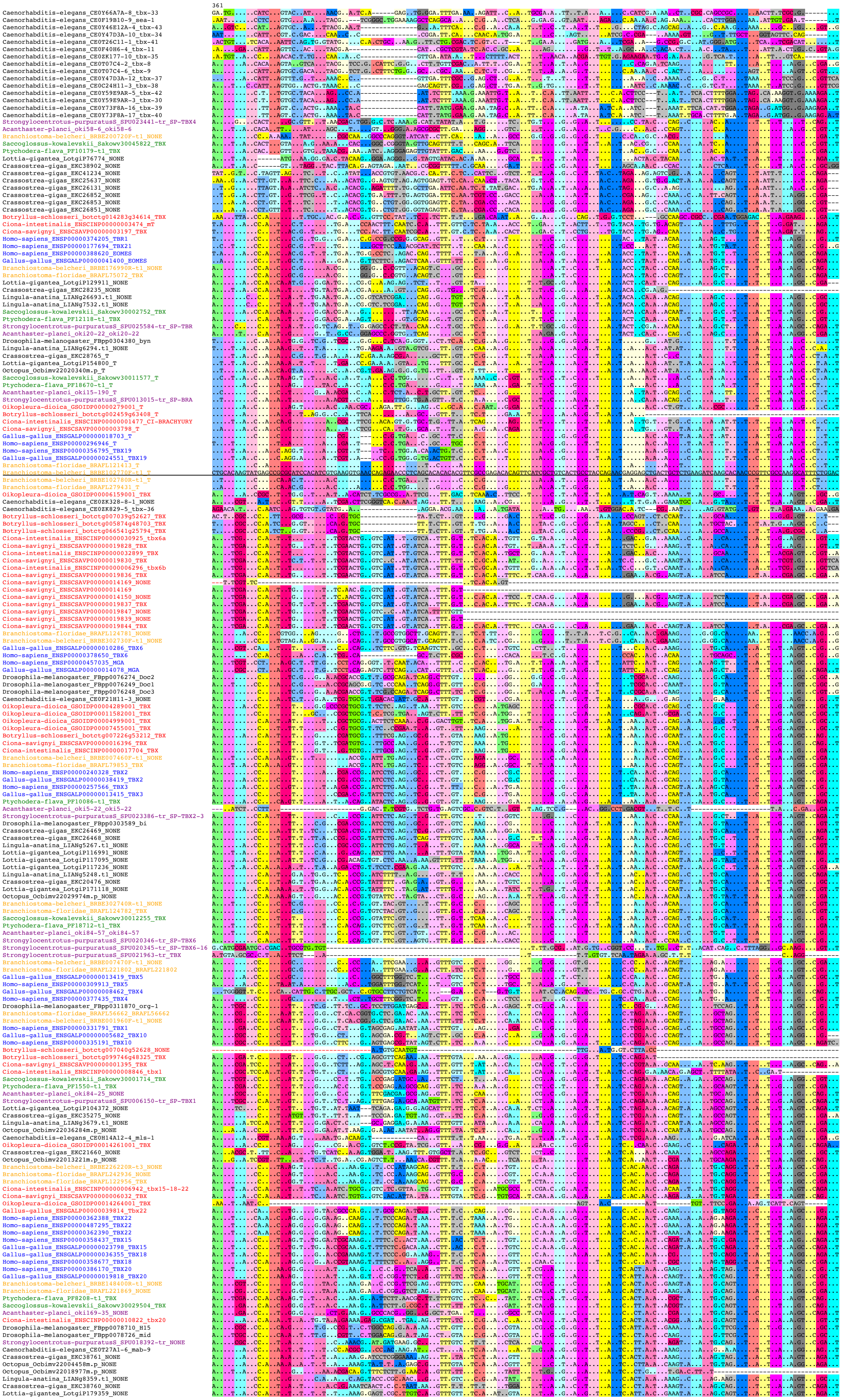

Additional file 1
(continued)
