# Supplementary material for: The chordate ancestor possessed a single copy of the Brachyury gene for notochord acquisition
Source: Zoological Lett. 2017 Mar 23;3:4. doi: 10.1186/s40851-017-0064-9 (PMC5363035; doi:10.1186/s40851-017-0064-9)

a. DNA dataset.

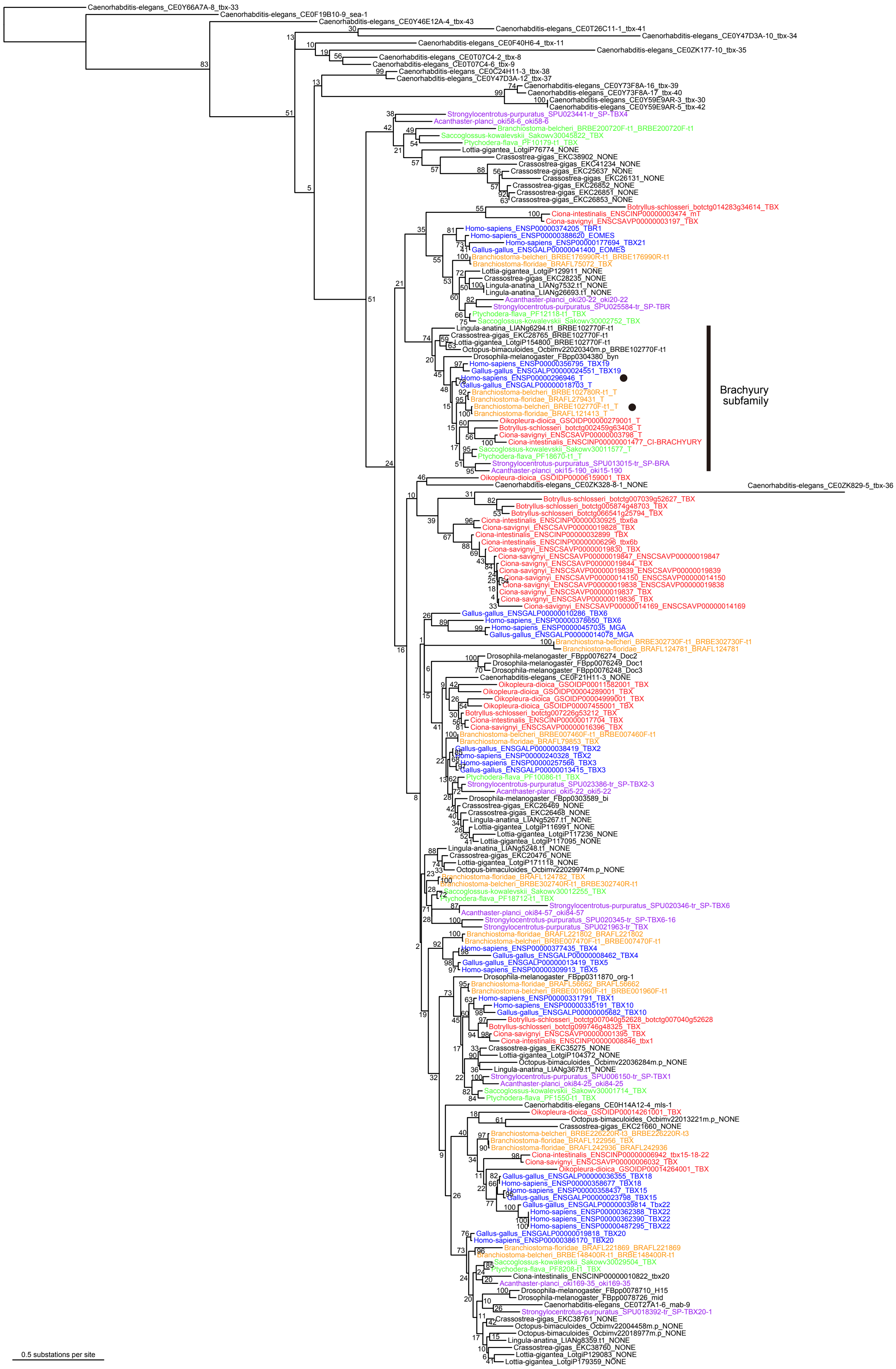

Additional file 2

b. Amino acid dataset.

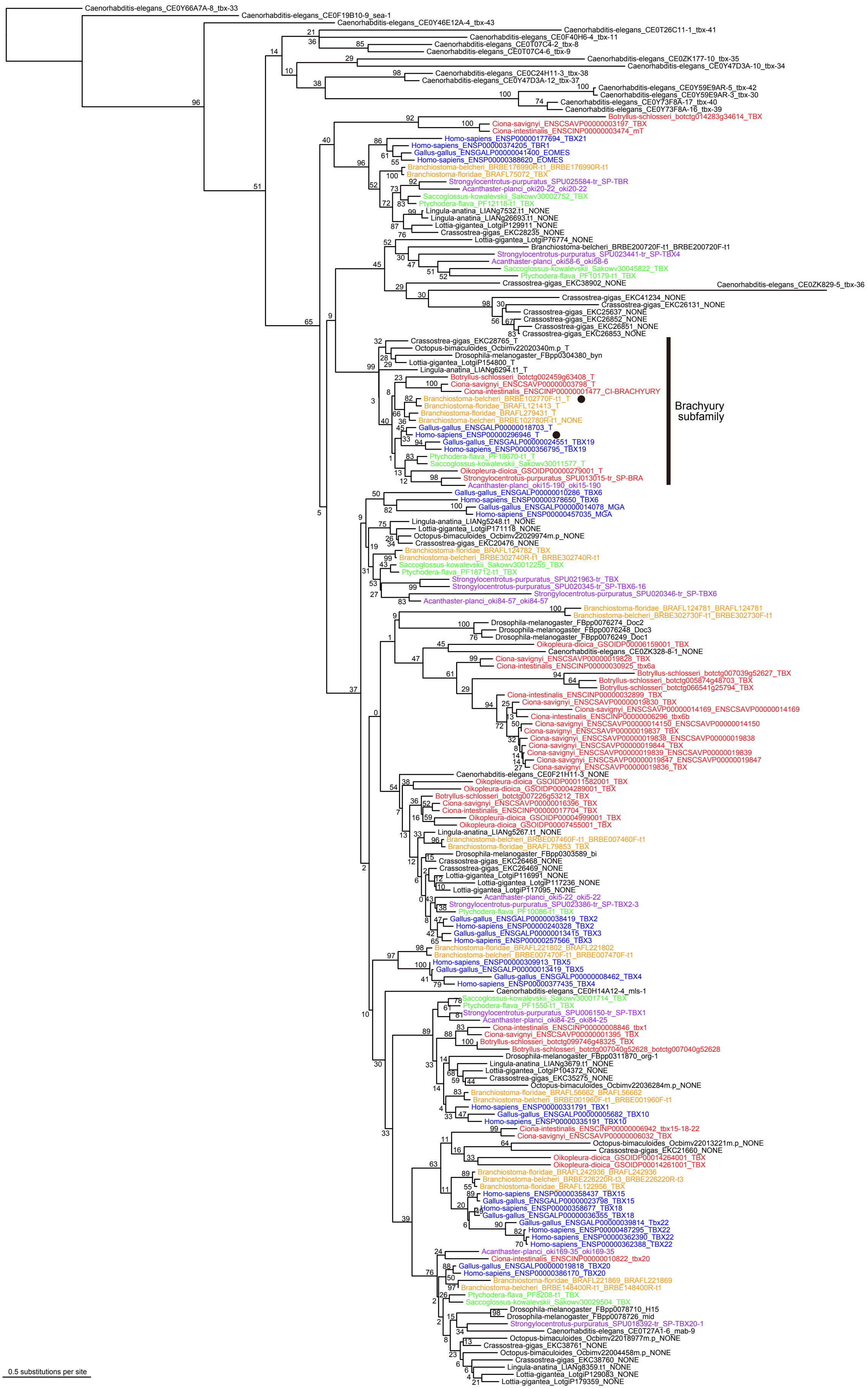

Supplement: Supplementary file 2 — Molecular phylogenies of T-box family members based on a DNA dataset comprising 350 unambiguously aligned sites (excluding 3rd codon positions) (a) and based on an amino acid dataset comprising 175 sites (b). In both trees, the Brachyury family consistently forms a distinct clade among T-box family members. The resulting tree obtained from reanalysis using only this portion is shown in Fig. 2 and Additional file 4. Query sequences used for the BLAST search are marked with black dots. (PDF 551 kb) [file 40851_2017_64_MOESM2_ESM.pdf]
