# Supplementary material for: The chordate ancestor possessed a single copy of the Brachyury gene for notochord acquisition
Source: Zoological Lett. 2017 Mar 23;3:4. doi: 10.1186/s40851-017-0064-9 (PMC5363035; doi:10.1186/s40851-017-0064-9)
Supplement: Supplementary file 3 — cDNA sequence alignment of Brachyury genes (2625 sites in total) analyzed in the phylogenetic analyses (Fig. 2 and Additional file 4). The alignment was constructed using the selected gene sequences from the estimated tree of T-box-containing genes (Additional file 2). Unambiguously aligned sites indicated by a 1 (on the top of the sequences, 1308 sites) were used for the analyses. (PDF 3214 kb) [file 40851_2017_64_MOESM3_ESM.pdf]

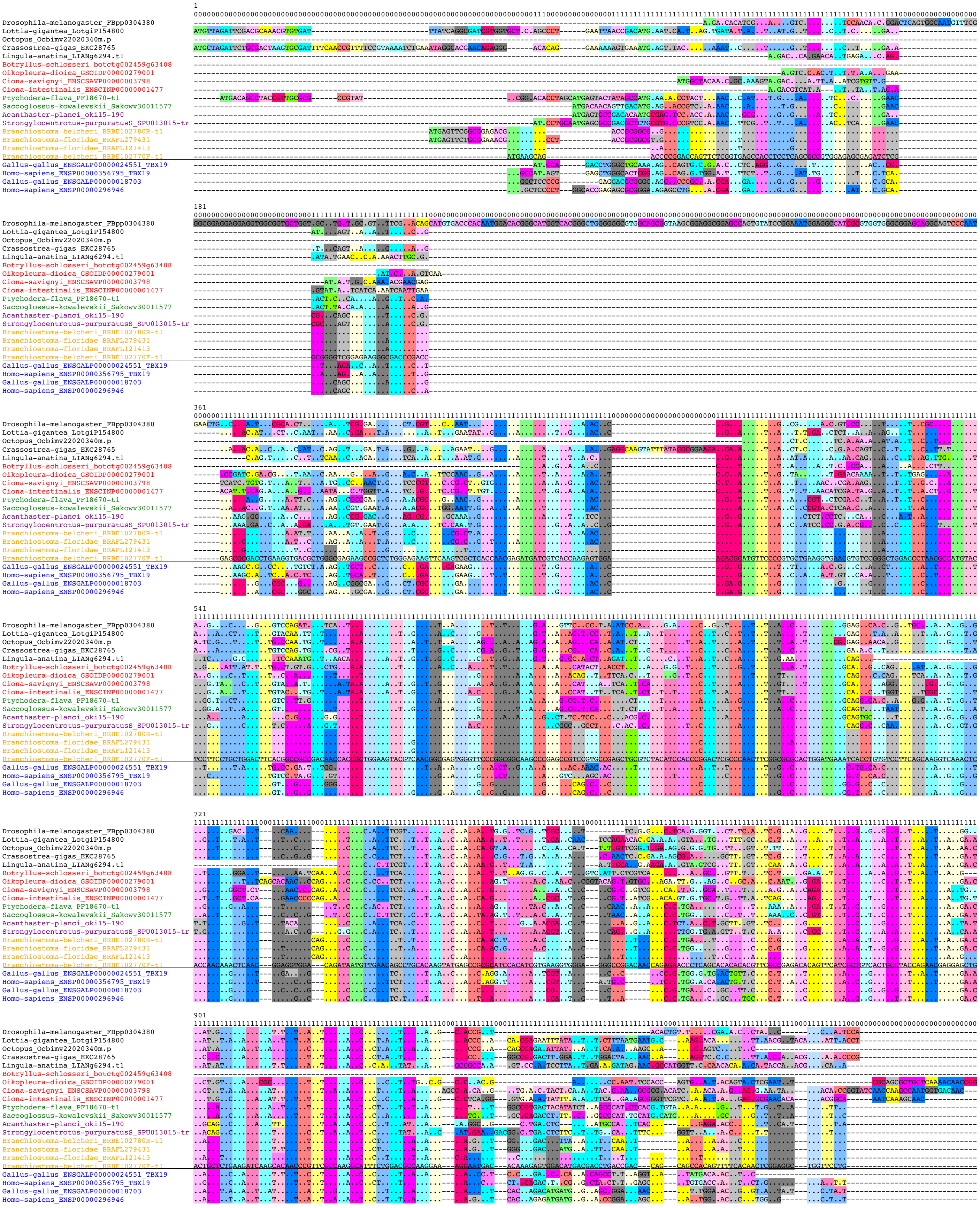

Additional file 3

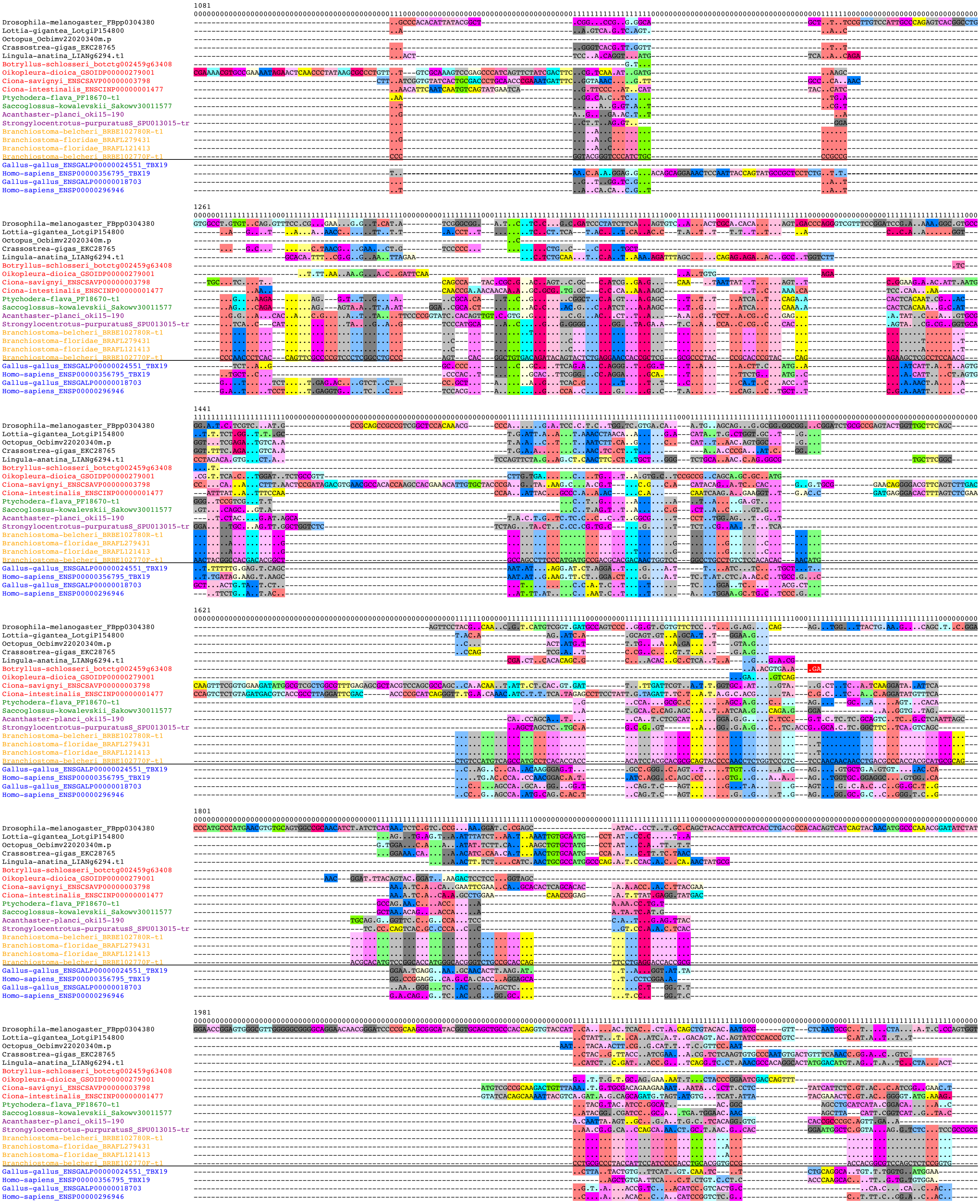

Additional file 3
(continued)

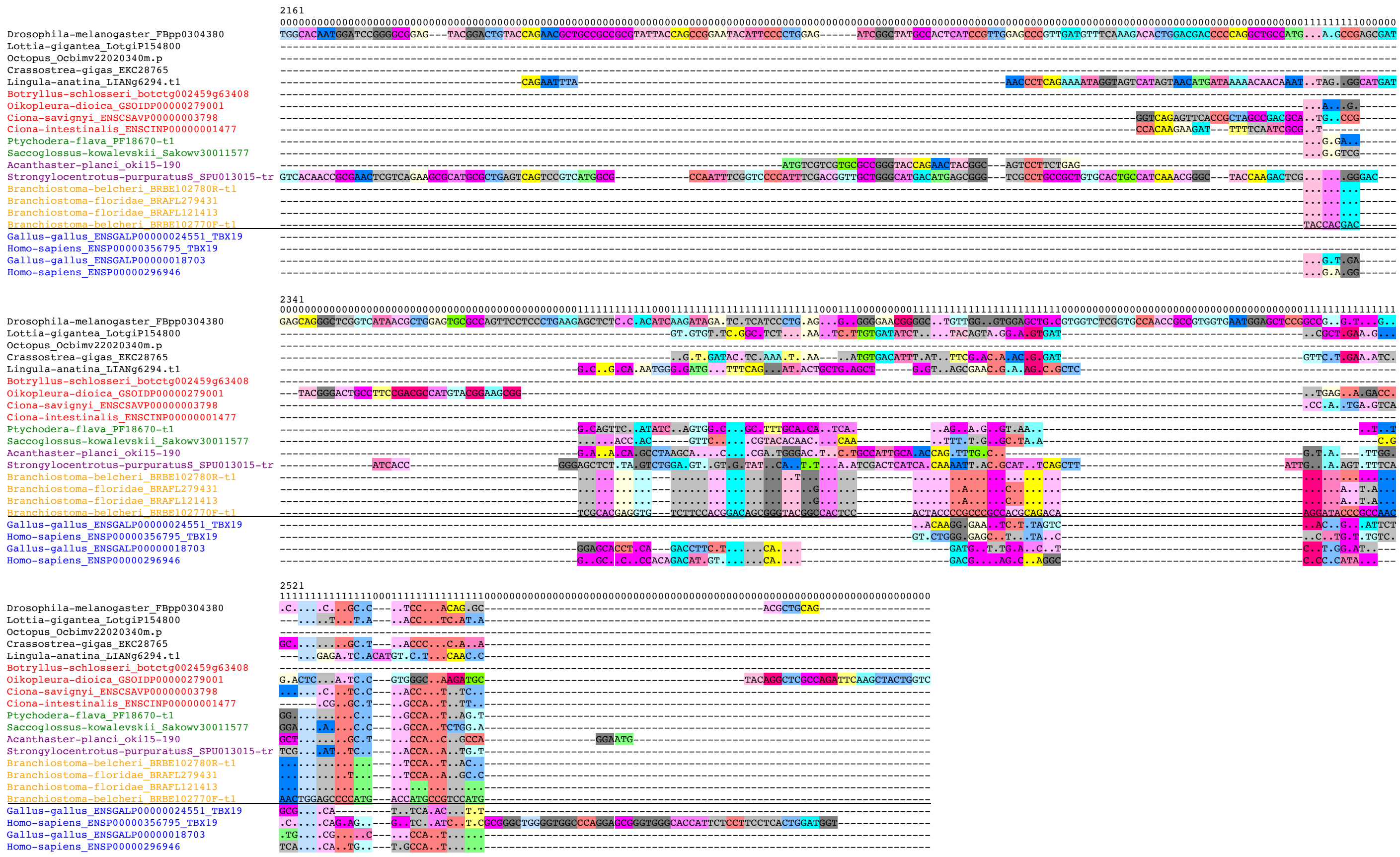

Additional file 3
(continued)
