# Supplementary figures and images for: The chordate ancestor possessed a single copy of the Brachyury gene for notochord acquisition
Source: Zoological Lett. 2017 Mar 23;3:4. doi: 10.1186/s40851-017-0064-9 (PMC5363035; doi:10.1186/s40851-017-0064-9)

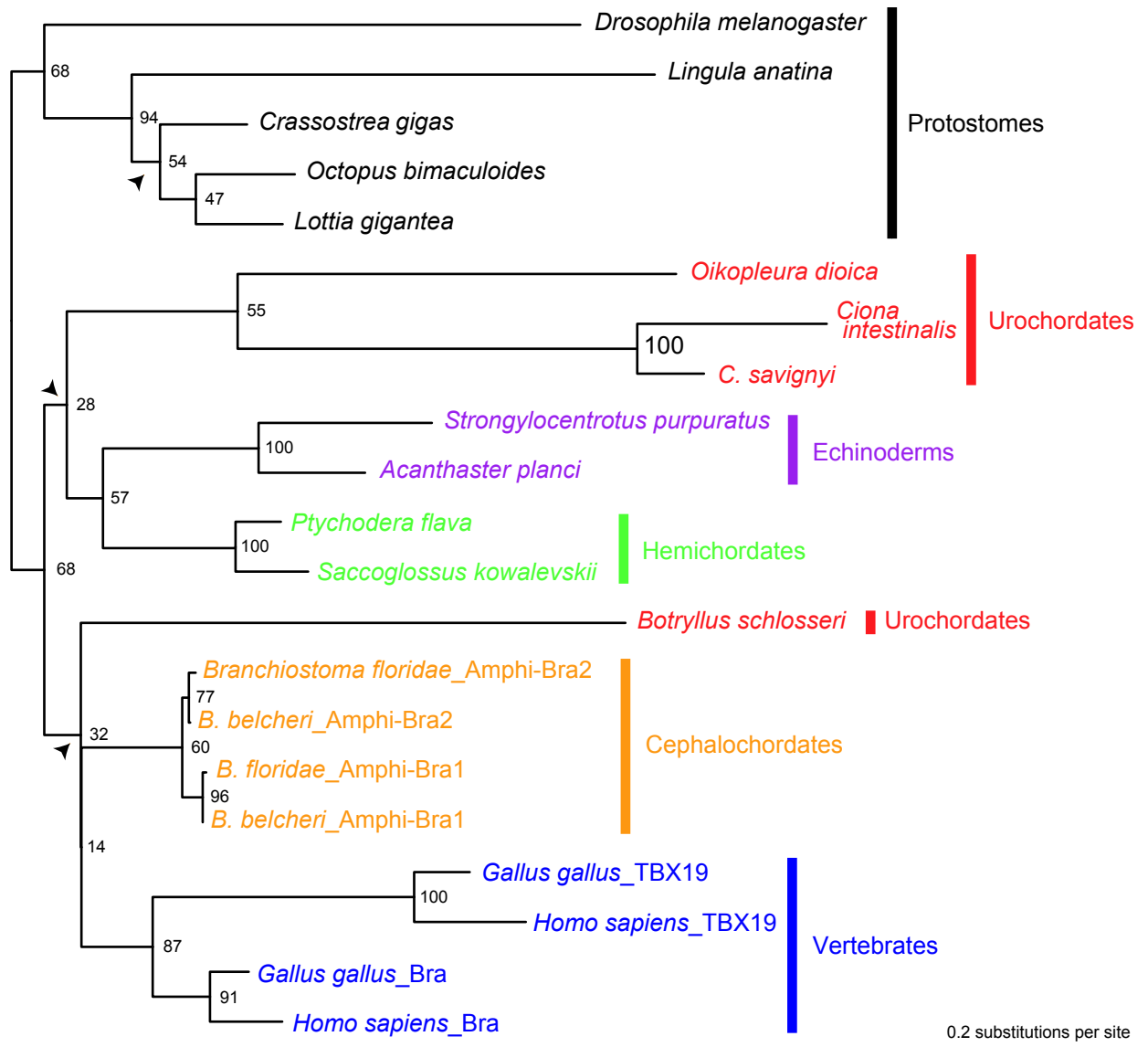

Supplement: Supplementary file 4 — Molecular phylogeny of Brachyury family members based on an amino acid dataset comprising 436 unambiguously aligned sites. Arrowheads indicate topological incongruities with the tree obtained from comparisons of nucleotides (Fig. 2). Probably due to the short length of the analyzed sequence, the Botryllus schlosseri (urochordate) gene was placed as a sister lineage of a clade comprising cephalochordate and vertebrate genes. (PDF 150 kb) [file 40851_2017_64_MOESM4_ESM.pdf]
