# Supplementary material for: The chordate ancestor possessed a single copy of the Brachyury gene for notochord acquisition
Source: Zoological Lett. 2017 Mar 23;3:4. doi: 10.1186/s40851-017-0064-9 (PMC5363035; doi:10.1186/s40851-017-0064-9)
Supplement: Supplementary file 5 — Conserved synteny around Brachyury in deuterostomes. (DOCX 125 kb) [file 40851_2017_64_MOESM5_ESM.docx]

**Additional file 5**

**Conserved synteny around *Brachyury* in deuterostomes**

**Major deuterostome lineages**

Sometimes synteny (Fig. 3) is similar among vertebrate species or ambulacrarians (e.g., sea urchin and acorn worms). For example, on chicken chromosome 3 and human chromosome 6, a set of genes has the following order: *RPS6KA2*, *MPC1*, *SET2D1*, *Brachyury*, *PDE10A*, *QKI* and *PACRG*. Less distinct synteny was evident between the sea urchin (*Strongylocentrotus purpuratus*) and two acorn worms (*Ptychodera flava* and *Saccoglossus kowalevskii*), UFL1 being located near *Brachyury*. In addition, some genes share a comparable or similar DNA sequence. This suggests that characterization of global synteny between echinoderms and hemichordates is important to understand the evolution of two clades from a common ambulacrarian ancestor.

**Urochordates**

In contrast to the synteny found in ambulacrarians or vertebrates, no conserved synteny was found among four species of tunicates (Fig. 3). In the case of two *Ciona* species, *C. intestinalis* and *C. savignyi*, scaffolds of each species ended at the *Brachyury*-containing region, making synteny analyses difficult. Scaffolds that contain *Brachyury* in *Oikopleura* and *Botryllus* provided better information for synteny analysis of *Brachyury* and neighboring genes. Even under such conditions, we could not find any syntenic presentation of homologous genes in genomic region containing *Brachyury*. This is presumably due to higher rate of molecular evolution and reorganization of urochordate genes (1).

**Cephalochordates**

Most important in the context of the present research, it is highly likely that the duplication of *Brachyury* occurred only in cephalochordates, as *Brachyury* is present in a single copy in the four other deuterostome taxa (Fig. 3). When *Brachyury*-containing scaffolds are compared between *B. floridae and B. belcheri*, three genes, *NOTUM*, *BVES*, and *SOCS3* were also duplicated in addition to *Brachyury* and the direction of duplication is not always the same, i.e. one is a tandem duplication on the same strand (*BVES* and *SOCS3*) and the other on a different strand (*Brachyury* and *NOTUM*). This genomic region also allowed duplication in amphioxus, although molecular mechanisms for the duplication remain to be explored in the future.

**Vertebrates**

Although synteny analysis among vertebrate genomes was complicated by the 2R-GWGD, it is evident that there are no scaffolds with duplicated *Brachyury* or its ohnolog, *Tbx19*, in the chicken or human genomes (Fig. 3). As mentioned above, *Brachyury* is present as a single copy on chicken chromosome 3 and human chromosome 6. No other T-box genes reside near *Brachyury*. This case can also be seen on chicken chromosome 1 and human chromosome 1, where *Tbx19* is located. However, *SFT2D*-related genes (*SFT2D1*/ *SFT2D2*) were located near *Brachyury* in all four vertebrate genomes. This partial conservation of synteny between regions including *Brachyury* and *Tbx19*, in addition to the monophyletic relationships of vertebrate *Brachyury* and *Tbx19* (Fig. 2), suggest that these genomic regions were duplicated during the 2R-GWGD.

Our results indicate that there is nothing in synteny around *Brachyury*, suggesting that no *Brachyury* duplication occurred in the common ancestor shared by cephalochordates and other chordates (Fig. 3).
